# Maturação somática e o relacionamento entre variáveis minerais ósseas e modalidades esportivas em adolescentes: estudo transversal: Somatic maturation and the relationship between bone mineral variables and types of sports among adolescents: cross-sectional study

**DOI:** 10.1590/1516-3180.2016.0270210217

**Published:** 2017-04-03

**Authors:** Ricardo Ribeiro Agostinete, Igor Hideki Ito, Han Kemper, Carlos Marcelo Pastre, Mário Antônio Rodrigues-Júnior, Rafael Luiz-de-Marco, Rômulo Araújo Fernandes

**Affiliations:** I MSc. Postgraduate Student, Postgraduate Program on Physical Therapy, Laboratory for Exercise Investigation (LIVE), Department of Physical Education, Universidade Estadual Paulista (UNESP), Presidente Prudente (SP), Brazil.; II MSc. Postgraduate Student, Postgraduate Program on Kinesiology, Laboratory for Exercise Investigation (LIVE), Department of Physical Education, Universidade Estadual Paulista (UNESP), Presidente Prudente (SP), Brazil.; III PhD. Emeritus Professor, Department of Occupational Health, EMGO+ Institute for Health and Care Research, VU University Medical Center, Amsterdam, Netherlands.; IV PhD. Associate Professor, Department of Physical Therapy, Universidade Estadual Paulista (UNESP), Presidente Prudente (SP), Brazil.; V MSc. Postgraduate Student, Laboratory for Exercise Investigation (LIVE), Department of Physical Education, Universidade Estadual Paulista (UNESP), Presidente Prudente (SP), Brazil.; VI PhD. Assistant Professor, Laboratory for Exercise Investigation (LIVE), Department of Physical Education, Universidade Estadual Paulista (UNESP), Presidente Prudente (SP), Brazil.

**Keywords:** Adolescent, Bone density, Growth and development, Sports, Puberty

## Abstract

**CONTEXT AND OBJECTIVE:**

Peak height velocity (PHV) is an important maturational event during adolescence that affects skeleton size. The objective here was to compare bone variables in adolescents who practiced different types of sports, and to identify whether differences in bone variables attributed to sports practice were dependent on somatic maturation status.

**DESIGN AND SETTING:**

Cross-sectional study, São Paulo State University (UNESP).

**METHODS:**

The study was composed of 93 adolescents (12 to 16.5 years old), divided into three groups: no-sport group (n = 42), soccer/basketball group (n = 26) and swimming group (n = 25). Bone mineral density and content were measured using dual-energy x-ray absorptiometry and somatic maturation was estimated through using peak height velocity. Data on training load were provided by the coaches.

**RESULTS:**

Adolescents whose PHV occurred at an older age presented higher bone mineral density in their upper limbs (P = 0.018). After adjustments for confounders, such as somatic maturation, the swimmers presented lower values for bone mineral density in their lower limbs, spine and whole body. Only the bone mineral density in the upper limbs was similar between the groups. There was a negative relationship between whole-body bone mineral content and the weekly training hours (β: -1563.967; 95% confidence interval, CI: -2916.484 to -211.450).

**CONCLUSION:**

The differences in bone variables attributed to sport practice occurred independently of maturation, while high training load in situations of hypogravity seemed to be related to lower bone mass in swimmers.

## RESUMO

**CONTEXTO E OBJETIVO:**

O pico de velocidade de crescimento (PVC) constitui importante evento maturacional durante a adolescência, afetando o tamanho do esqueleto. O objetivo do estudo foi comparar variáveis ósseas em adolescentes praticantes de diferentes modalidades esportivas, bem como identificar se diferenças nas variáveis ósseas atribuídas à prática esportiva são dependentes do estado da maturação somática.

**DESENHO E LOCAL:**

Estudo transversal, Universidade Estadual Paulista (UNESP).

**MÉTODOS:**

O estudo foi constituído por 93 adolescentes (12 a 16,5 anos), divididos em três grupos: grupo sem envolvimento esportivo (n = 42), futebol/basquete (n = 26) e natação (n = 25). A densidade e conteúdo mineral ósseo foram mensurados utilizando absortiometria de raio-x de dupla energia e a maturação somática foi estimada através do uso do PVC. Informações sobre volume de treinamento foram fornecidas pelos treinadores.

**RESULTADOS:**

Adolescentes com idade tardia no PVC apresentaram maiores valores de densidade mineral óssea em membros superiores (P = 0,018). Após ajustes por variáveis de confusão, como a maturação somática, os nadadores apresentaram menores valores de densidade mineral óssea em membros inferiores, coluna e corpo inteiro. Apenas a densidade mineral óssea de membros superiores foi similar entre os grupos. Existiu relação negativa entre conteúdo mineral ósseo de corpo inteiro e tempo de treino semanal (β: -1563.967; 95% intervalo de confiança, IC: -2916.484 a -211.450).

**CONCLUSÃO:**

As diferenças em variáveis ósseas atribuídas à prática esportiva ocorrem independentemente da maturação, enquanto elevada quantidade de treinamento em situações de hipogravidade parece estar relacionada com menor massa óssea em nadadores.

## INTRODUCTION

Osteoporosis constitutes a widespread disease among the elderly population and it is associated with high economic costs.^1^^,^^2^ Although less common in pediatric populations, development of osteoporosis has been linked to peak bone mass gained during early life.^3^^,^^4^ There is a natural decrease in bone mass during later life and, therefore, adolescents who present lower peak bone mass have an increased likelihood of developing osteoporosis during adulthood.^3^^,^^4^ Consequently, early life has been highlighted as a critical period for development of osteoporosis during adulthood.^5^ Hence, variables relating to peak bone mass during childhood and adolescence have been widely investigated and several variables have been pointed out as potential determinants of modifications to peak bone mass, such as: nutritional factors, genetics and practicing physical activity.^3^^,^^4^^,^^5^

Regarding physical activity, its practice stimulates release of hormones relating to higher rates of bone formation.^6^^,^^7^ In addition, the physical load on bone structure that is generated by exercise stimulates its turnover.^7^ Several sports cause high training load during practice and thus act positively on bone accrual during growth, such as soccer, tennis and rugby.^8^^,^^9^^,^^10^^,^^11^ However, the effects on bone mineral variables caused by sports participation performed in hypogravity during adolescence still remain unclear,^10^^,^^12^ mainly because previous studies failed to control for the burden of important potential confounders in early life, such as training load and biological maturation.^12^

In terms of bone mass gain, the pubertal period is responsible for significant accrual of bone mass in both boys and girls.^13^ Peak height velocity (PHV) is an important maturational event that occurs during adolescence, significantly increasing the skeleton size.^13^ PHV precedes the peak bone mass accrual, thus denoting a period of potential risk of fractures,^13^^,^^14^ during which skeletal mass does not accompany the increase in skeleton size. On the other hand, the impact of this dissociation between linear growth and bone mass gain on the recognized osteogenic effect caused by exercise is still unclear.

## OBJECTIVE

Therefore, the purposes of this study were:

1. To compare bone mineral variables in adolescents according to different sports; and
2. To identify whether the potential differences in bone mineral variables attributed to sports participation are dependent on somatic maturation status.

## METHODS

### Design, setting and ethics

This was a cross-sectional study that formed part of a larger cohort study entitled “*Practice of different sports and bone mass gain in adolescents: 9-month cohort study*”, which was conducted from October 2013 to July/August 2014. The information presented in this study concerns data collected at the baseline. Adolescents were recruited from three public schools and three sports clubs that specialized in soccer, basketball and swimming. This study was previously approved by our institution’s ethics board (CAAE; no. 02891112.6.0000.5402).

### Sample

The sample size estimation was made using an equation based on analysis of variance (ANOVA), which took into account a minimum difference for whole-body bone mineral content (BMC) of 89 grams between the control and sports groups,^15^ a standard deviation of 37 g for three independent groups (no-sport, swimming and soccer groups), power of 80% and alpha of 5%. The final sample size was estimated as a minimum of 12 adolescents per group and, therefore, a minimum of 36 adolescents was required.

### Participants

The following inclusion criteria were adopted:

1. Chronological age between 11 and 17 years old;
2. A minimum of six months of practice (swimming, soccer or basketball) or absence of participation in any organized sports over the previous six months (no-sport group);
3. No use of medication that could affect bone metabolism; and
4. Prior authorization from the coach and parents to take part in the study and presentation of a signed consent form.

Initially, 190 adolescents of ages ranging from 11 to 17 years old were contacted (74 in the no-sport group and 116 athletes).

Adolescents in the no-sport group were excluded in the following situations: [1] practice of organized sport within the previous six months; or [2] engagement in recreational sports activities on more than two days per week. Adolescents involved in organized sports were excluded in the following situations: [1] less than six months of practice in the current sport; or [2] engagement in more than one sport. For statistical analysis, impact sports (soccer and basketball) were combined into one group.

The swimmers and soccer players participated in competitions at national level, while the basketball players participated in regional-level competitions. Their coaches provided data on their weekly training load over the previous six months (minutes/week [min/wk]). The athletes reported their previous engagement time (in months) and the age at which they started practicing their current sport.

### Anthropometry

Body mass was measured using an electronic scale (Filizola model PL 150; Filizola Ltda., Brazil). Total height (i.e. height when standing upright) and seated height were measured using a stadiometer (Sanny model; American Medical of Brazil Ltda., Brazil) and leg length was calculated by subtraction of seated height from total height. A single trained technician made all the measurements on the subjects during a visit that they made to the laboratory.

### Bone mineral variables

Bone mineral density (BMD, in g/cm^2^), BMC (in grams), body fat (in percentages) and fat-free mass (FFM; in kilograms) were assessed using a dual-energy x-ray absorptiometry scanner (Lunar DPX-NT; General Electric Healthcare, Little Chalfont, Buckinghamshire, UK) with Lunar software (version 4.7) (GE Medical Systems). The scanner quality was tested by a trained researcher prior to each day of measurement, following the manufacturer’s recommendations. The participants wore light clothing, without shoes, and remained in the supine position on the machine (approximately 15 minutes). BMD measurements were made for:

1. Head (head and neck);
2. Upper limbs;
3. Lower limbs;
4. Spine; and
5. Whole body.

The proportion of each of these body segments as a percentage of whole-body BMC was calculated for the head, lower limbs, upper limbs and trunk (including rig cage, spine and pelvis), as follows: [BMC-body segment * 100]/overall BMC. The precision of the machine in terms of coefficient of variation was 0.66% (n = 30 subjects not involved in this study) and all scans were carried out in a temperature-controlled laboratory at the university.

### Peak height velocity

The measurements of body mass, height, seated height and leg length were used to calculate maturity offset, which denotes the time (years) from/to PHV.^16^ The sample was stratified according to PHV as follows: (i) “on time” (boys: 13.4-14.8 years; and girls: 11.8-13.0); and (ii) “late” (boys: > 14.8 years; and girls: > 13.0 years), and “early” (boys: < 13.4 years; and girls: < 11.8 years).^17^

Maturity offset for boys (in years) = -9.236

+ (0.0002708 * (leg length * seated height))

+ (-0.001663 * (age * leg length)) 

+ (0.007216 * (age * seated height))

+ (0.02292 * (body mass/height *100))

Maturity offset for girls (in years) = -9.376

+ (0.0001882 * (leg length * seated height))

+ (0.0022 * (age * leg length))

+ (0.005841* (age * seated height))

+ (0.002658 * (age * body mass))

+ (0.07693 * (body mass/height *100))

### Statistical analysis

The descriptive statistics comprised the mean, standard error of the mean (SEM) and 95% confidence interval (95% CI). Training load (minutes per week) presented nonparametric distribution and was therefore analyzed after logarithmic transformation. Linear regression was used to analyze the relationship between training load and bone mineral variables, adjusted for chronological age, sex, biological maturation, height, FFM and duration of previous engagement in the sport. Analysis of covariance (ANCOVA) was used to compare mean differences according to sports, controlled for potential confounders (chronological age, sex, biological maturation, height, FFM and duration of previous engagement in the sport), and to generate estimated means and SEM. In all the ANCOVA models, homogeneity of variance was assessed using Levene’s test. The associated magnitude effect was determined by means of effect size correlations (ES-r). This effect size was estimated using the square root of the ratio between the F-value squared and the difference between the F-value squared and degrees of freedom. Coefficients were interpreted as follows: trivial (r < 0.1), small (0.1 > r < 0.3), moderate (0.3 > r < 0.5), large (0.5 > r < 0.7), very large (0.7 > r < 0.9), nearly perfect (r > 0.9) and perfect (r = 1).^18^ Statistical significance (P-value) was set at P < 0.05 and the statistical software BioEstat (version 5.0) was used to perform analyses.

## RESULTS

For this study, after checking for compliance with the inclusion criteria, only 93 eligible adolescents were contacted. They were at six different locations, including public/private schools and sports clubs: no-sport group (n = 42) and sport groups (n = 51; 25 swimmers, 18 soccer players and 8 basketball players). The sample thus selected was composed of adolescents of both sexes equally (58.1% boys and 41.9% girls; χ^2^ = 2.419; P = 0.120) with ages ranging from 12 to 16.5 years.

**Figure 1:**
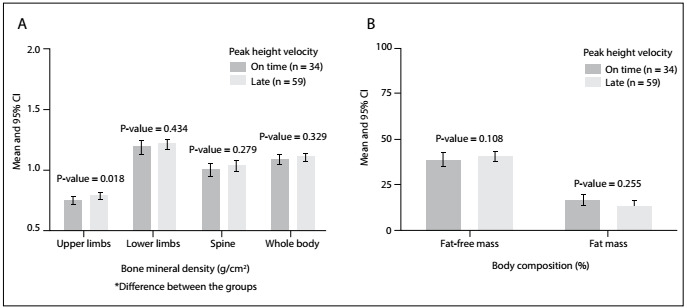
Bone mineral density and body composition in adolescents stratified according to peak height velocity.

Table 1 presents descriptive results according to groups of sport practice. There were significant differences for height, fat mass (%) and fat-free mass (kg) in the no-sport group compared with the soccer/basketball group and swimmers. Regarding bone variables (bone mineral density and bone mineral content), we found significant differences between the groups regarding the upper limbs, lower limbs and whole body. After stratification according to PHV, adolescents with late PHV presented higher BMD in the upper limbs, while the values for fat mass and FFM were similar (Figure 1).

**Table 1:**
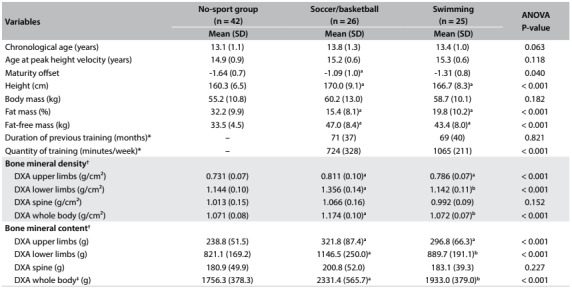
Characteristics of adolescents practicing different sports (12 to 16.5 years of age)

*Values presented as median and interquartile range; ^†^Variables without adjustment for sex, age, peak height velocity, duration of previous engagement, height and fat-free mass; ^‡^without head; SD = standard deviation; DXA = dual energy x-ray absorptiometry; ^a^P-value < 0.05 compared with control; ^b^P-value < 0.05 compared with soccer/basketball; ANOVA = analysis of variance.

After adjustments for confounders, such as somatic maturation, swimmers had lower values for BMD in their lower limbs, spine and whole body, compared with the no-sport group and basketball players. Only BMD in the upper limbs was similar between the groups (Table 2). The somatic maturation (PHV) presented a trivial effect size in all BMD variables: upper limbs (ES-r 0.000; P = 0.840), lower limbs (ES-r 0.030; P = 0.110), spine (ES-r 0.004; P = 0.582) and whole body (ES-r 0.004; P = 0.545). BMD in the lower limbs and whole body were marginally significant in the soccer/basketball group, compared with the swimmers and no-sport group.

**Table 2:**
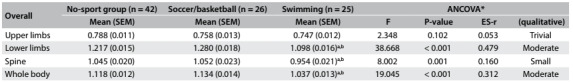
Bone mineral density among adolescents, stratified according to sports participation

*Adjusted for sex, age, peak height velocity, duration of previous engagement, height and fat-free mass; ANCOVA = analysis of covariance; SEM = standard error of the mean; ES-r = Eta-squared correlations (r-Hopkins); ^a^P-value < 0.05 compared with control; ^b^P-value < 0.05 compared with soccer/basketball.

In the whole sample, the percentage of BMC accounted for by the individual’s head in comparison with the whole-body BMC was 18.5% (range: 12.3% to 27.7%), upper limbs 11.4% (8.8% to 14.7%), lower limbs 38.6% (31.4% to 45.8%) and trunk 31.3% (24.1% to 37.5%). Swimmers’ heads represented a higher percentage of whole-body BMC, compared with the soccer/basketball group (P = 0.002). On the other hand, the lower limbs of the soccer/basketball group represented a higher percentage of whole-body BMC, compared with the swimmers (P < 0.001). Swimmers’ upper limbs presented a higher percentage of whole-body BMC, compared with the soccer/basketball group (P < 0.001) and the control group (P = 0.010) (Table 3). Somatic maturation (PHV) also showed a trivial effect size in all variables (body sites) that constituted overall BMC: head (ES-r 0.000; P = 0.987), upper limbs (ES-r 0.000; P = 0.873), lower limbs (ES-r 0.004; P = 0.568) and trunk (ES-r 0.006; P = 0.475) (Table 3).

**Table 3:**
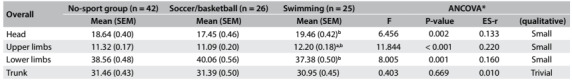
Estimated mean percentage participation (of each body segment) in overall BMC (g), among adolescents according to sports participation.

*Adjusted for sex, age, peak height velocity, duration of previous engagement, height and fat-free mass; ANCOVA = analysis of covariance; SEM = standard error of the mean; ES-r = eta-squared correlations (r-Hopkins); BMC = bone mineral content; ^a^P-value < 0.05 compared with control; ^b^P-value < 0.05 compared with soccer/basketball.

Finally, there was a negative relationship between whole-body BMC and weekly training time (β: -1563.967; 95% CI: -2916.484 to -211.450) (Table 4).

**Table 4:**
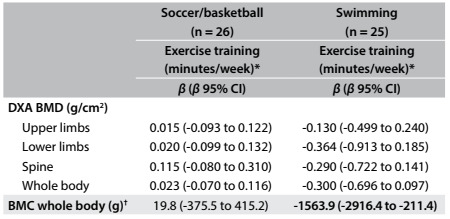
Linear regression (controlled for sex, CA, AHPV, FFM, height and duration of previous training (years) between bone mass and time spent training, among adolescents according to sports

*Numerical variable after logarithmic transformation; ^†^without head; 95% CI = 95% confidence interval; DXA = dual energy x-ray absorptiometry; CA = chronological age; APHV = age at peak height velocity; FFM = fat-free mass.

## DISCUSSION

This cross-sectional study found that swimmers presented lower bone density than the no-sport group and the group of adolescents engaged in impact sports, independently of somatic maturation.

In the present study, sports participation was related to higher BMD (upper limbs) in adolescents who reached PHV at a late age, but not in adolescents classified as on time. In a systematic review^19^ composed of 22 studies, Specker et al. found that exercise protocols targeting bone mass accrual seemed to be more effective in samples composed of pre-pubertal adolescents. The period of maturity offset between -2.0 and +2.0 was characterized by significant linear growth and BMC accrual.^13^ Independently of sex, the two years between PHV -1.0 and + 1.0 encompass the peaks of testosterone, estradiol and insulin-like growth factor-1 (IGF-1) levels.^20^ Similarly, participation in sports acts towards releasing anabolic hormones, such as IGFBP-3 and testosterone.^21^ The anabolic effect promoted by sports participation might be improved through maturational events occurring in the years before and after PHV, and adolescents reaching PHV at a late age are more likely to improve their bone health.

On the other hand, even though significantly boosted by maturation events, the osteogenic effect linked to physical exercise occurs during periods of lower hormonal activity. For instance, Ferry et al.^10^ found that 12 months of soccer practice enhanced bone mass and geometry, even in post-pubertal adolescents, while this effect was not observed in swimmers. Therefore, the improvement in bone mass through sports participation seems also to be observed in later adolescence when most other maturational events have been completed.

In our sample, the percentage of participation of the head in the whole-body bone mass was higher in swimmers than in soccer players. Regarding this issue, there have been divergences in the scientific literature. Gómez-Bruton et al.^12^ suggested that increased bone mass at other specific sites directly stressed by sports participation, even without a clear biological pathway, is related to lower skull bone mass. In contrast, our findings showed that even after controlling for potential confounders, not only did swimmers have a higher percentage of overall BMC in the skull (no difference between the soccer/basketball and no-sport groups), but also they had a higher percentage of overall BMC in their upper limbs than the soccer/basketball players and the control group. In the soccer/basketball group, there was no apparent decrease in skull bone mass due to sports participation; rather, it presented a lower percentage of whole-body BMC due to the additional bone mass gained at other sites that were directly stimulated by sports participation.

In the present study, the lower values for bone variables in swimmers, compared with soccer players, could be explained by the fact that swimming practice is performed in situations of hypogravity, characterized by lack of mechanical load and lower osteogenic stimulus.^12^ Among swimmers, greater weekly quantity of training hours was related to lower BMC in the whole body (excluding the head). Long periods of training performed under conditions of hypogravity and the inflammatory response to exercise might support our findings. Swimmers presented the highest numbers of weekly training hours (17.7 ± 3.5 h/week), which accounted for 15.8% (95% CI: 14.5-17.1) of their wakeful time during the week (assuming 8 hours of sleep per night in a period of 24 hours) and the longest duration of previous practice in the sport (69 ± 40 months). Ferry et al. identified lower numbers of weekly training hours among female swimmers (around 10 hours), but the sample was not composed of swimmers competing at national level (as observed in the present study).^10^

Moreover, swimming practice encompasses periods of training at high-intensity^21^ that are related to increased inflammation.^22^ Inflammatory marker levels increased by high-intensity exercise have been linked to lower action of growth hormone and IGF-1 in adolescents.^23^ Therefore, even without physiological measures relating to intensity, it is feasible to believe that the combination of prolonged inflammatory responses and large quantities of high-intensity training^23^ under conditions of hypogravity might explain the negative relationship observed between weekly training hours and BMC among swimmers.

However, in the present study, bone mineral density in the upper limbs was not reduced among swimmers, compared with the other groups. This result can be explained by the fact that swimming movements that generate propulsive forces are concentrated in the upper limbs, especially in the freestyle stroke.^24^ However, more studies are needed to corroborate and strengthen this finding.

The limitations of this study need to be recognized. The cross-sectional design constitutes a limitation because it does not allow statements of causality, although this limitation will in the future be overcome through follow-up measurements on this cohort. The lack of female participation in the impact sport group (basketball and soccer) is an important limitation considering that the maturational process and the effect of sports are different between the sexes. Therefore, it is relevant to consider that the differences found in our study according to sports groups may have been overestimated due to the imbalance of sex distribution. The age range in our study also constitutes a limitation, mainly because PHV has higher precision when applied to adolescents with ages ranging from 11 to 15 years old. The absence of nutritional variables also should be considered in future studies (e.g. calcium and vitamin D intake) because of their effect on bone formation. Finally, the effect of some hormones (e.g. testosterone, estradiol and growth hormone) and inflammatory markers need to be taken into account in future studies.

## CONCLUSION

In summary, the differences in bone variables attributed to sports participation occurred independently of maturation, while high training loads under conditions of hypogravity appear to be related to lower bone mass in swimmers.
